# Effects of Fruit Storage Temperature and Time on Cloud Stability of Not from Concentrated Apple Juice

**DOI:** 10.3390/foods11172568

**Published:** 2022-08-25

**Authors:** Haifen Wang, Junwei Yuan, Lan Chen, Zhaojun Ban, Yanli Zheng, Yuqian Jiang, Yunbin Jiang, Xihong Li

**Affiliations:** 1 State Key Laboratory of Food Nutrition and Safety, College of Food Science and Engineering, Tianjin University of Science and Technology, Tianjin 300457, China; 2 Tianjin Gasin-DH Preservation Technologies Co., Ltd., Tianjin 300300, China; 3 Shanxi Fruit Industry Cold Chain New Material Co., Ltd., Tongchuan 727100, China; 4 School of Biological and Chemical Engineering, Zhejiang University of Science and Technology, Hangzhou 310023, China

**Keywords:** ‘Fuji’ apples, storage conditions, freshly squeezed juice, cloud stability, particle size, not from concentrate

## Abstract

Apple juice that is designated ‘Not from concentrated’ (NFC) is now increasingly popular with consumers due to its unique taste and rich nutritional value. However, layered precipitation and instability have emerged as serious technical problems that restrict the viability of the NFC apple juice industry. This study researched the influence of water-cored ‘Fuji’ apple fruit storage under different temperatures (0, 20 °C) and times (0, 9, 18, 30, 60 days) on the turbidity stability of NFC apple juice. Changes in the physicochemical properties (juice yield, pH, total soluble solids and titratable acid), turbidity stability (turbidity and particle size) and precipitation sensitive substances (insoluble starch, total phenolics, soluble protein and pectin) of NFC apple juice were determined, combined with the respiratory rates and ethylene release of apples, in order to study post-harvest regulation and control of processed fruit. Results indicated that fruit storage temperature and time significantly guided the turbidity stability of NFC apple juice. As a typical respiratory climacteric fruit, apple fruit stored 45 days at 0 °C and 15 days at 20 °C gained the best juice stability, respectively. This is basically consistent with the respiratory peak of fruit when processing raw materials. During the post-ripening process, the insoluble starch in apple gradually hydrolyzed into fructose and glucose, while total phenolics diminished and water-soluble pectin content increased. On the other hand, the amounts of pectin, soluble protein and phenolics in fruit juice declined as the fruit aged in the late storage period (stored 75 days at 0 °C and 40 days at 20 °C). Meanwhile particle size became larger and the turbidity stability of cloudy juices also decreased. This study’s results will provide a sound theoretical basis for improving the turbidity stability of NFC apple juice by regulating the physiological state of processed raw materials.

## 1. Introduction

In the last few decades, with the increase in product range and people’s enhanced awareness of nutrition and health, consumers now demand high-quality food. NFC juice, i.e., freshly squeezed juice, has garnered more attention because it retains most of the original flavor and taste of juice, and meets people’s needs for healthy, natural and safe food [1,2]. NFC apple juice is one of the most important apple deep-processing products, which can effectively solve the problem of post-harvest apple surplus and increase the added value of apple products [3]. Nowadays, water-cored apples are the preference of many consumers, especially in Asia, due to its crystalline, water-soaked appearance around the core, as well as unique pineapple-like flavor [4,5]. Consumers now think that freshly squeezed cloudy water-cored apple juice is better for their health due to its higher levels of bioactive compounds than common apple juice, such as sorbitol, flavonols, flavanols, folic acid, dihydrochalcone, etc. [6,7].

During cloudy juice processing, coarse particles settle out shortly after juice extraction. The main factors affecting the cloudy stability of apple juice are firstly interaction between molecules, and secondly the aggregation of particles in the juice [8]. Precipitated particles mainly contain polyphenols, protein and pectin [9], and insoluble starch is one of the main reasons for the precipitation of apple juice [10]. The tiny cloud particles appear to be cell fragments that may originate from the cell membrane/cell wall of fruits [11]. These are relatively stable fine cloud particles composed of protein, polysaccharides, lipids and polyphenols [12]. There does appear to be a correlation between cell membrane/cell wall fragments and colloid-dissolved macromolecules, and naturally adsorbed pectin is an important aspect of cloud stability [13]. Zhu et al. [14] believed that there was no correlation between the particle size and soluble protein of apple juice. Processing and storage have little effect on the particle size, acidity and sugar content of juice [15]. It is found that cloud stability of juice was strongly correlated with chelator-soluble pectin in the research of Pan et al. [16].

Although many studies have focused on the quality and stability of NFC juice, they mainly compare different processing methods and storage periods [15,16,17]. Only a few studies have been published on the processing characteristics of raw materials. In fact, the nutritional components of apple juice depend on the genotype, environmental conditions and maturity of the fruit [18,19]. In addition, in actual production scenarios, raw materials used for juicing mostly originate from stored apples. With the extension of storage time, gases such as ethylene, carbon dioxide, etc., will accumulate continuously, and apples will gradually post-ripen, even aging [20]. However, research on the stability of NFC apple juice during raw material storage has not been reported.

**Figure 1:**
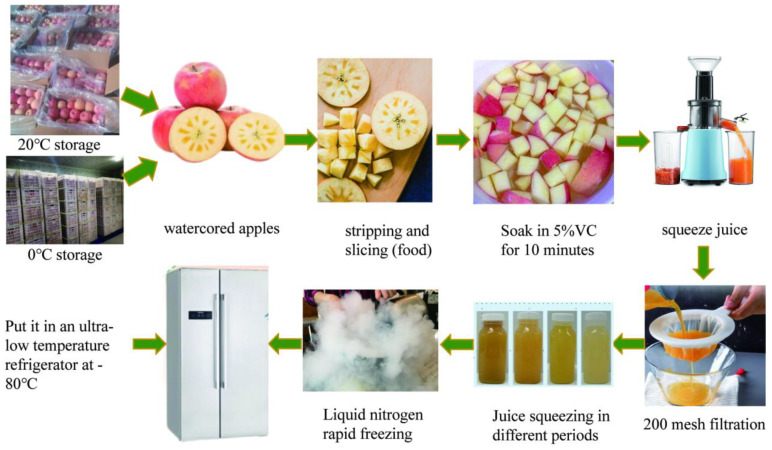
Pre-treatment and juicing process of water-cored ‘Fuji’ apple samples.

Therefore, in the present study, we chose water-cored ‘Fuji’ apples from AKsu in Xinjiang province in China as the research object. After the fruit were harvested on November 1st 2021, they were subjected to storage at 0 °C for 90 days and 20 °C for 45 days, respectively. As the most important index to reflect the physiological state of fruits, a part of the fruits was fixed for the determination of respiratory rates and endogenous ethylene release. With the extension of storage period at different temperatures, different pre-treated fruits were processed into juice for evaluation (Figure 1). The dynamic determination of juice yield, pH, total soluble solids (TSS), titratable acid (TA), turbidity, particle size, insoluble starch, total phenolics, soluble protein and pectin were carried out during whole storage. This investigation is important to study the stability of NFC apple juice, and provide a theoretical basis for improving the turbidity stability of NFC apple juice from the perspective of processing raw materials.

## 2. Materials and Methods

### 2.1. Plant Materials and Treatments

‘Fuji’ (Malus domestica) apples were harvested at the experimental orchards of HongQipo farm in Aksu (80°20′ E, 41°28′ N, Altitude 1105.3 m). Aksu is the most well-known apple cultivation region of China, and it is located at the foot of the Tian Shan mountain range in the west of China. The plant and row spacing of apple trees were 3 m and 4 m, respectively. Fruit were harvested on 1 November 2021 and sorted for uniform size and color, and then divided into two groups. Our treatments included: (1) fruit placed directly into plastic bags (3 μm thick) were stored at room temperature (20 °C); and (2) fruit pre-cooled to remove field heat followed by storage at 0 °C.

### 2.2. Fruit Respiration Rates and Ethylene Release Measurement

The respiration rates (RRCO_2_) were determined using a static respirometer method as described by Schnuerer et al. [21]. Analyses were carried out twice using a sample of five apples every 5 days during storage. After determining weight and diameter, individual fruits of five replicates were enclosed separately in a 4 L-tank glass jar for 0.5 h at 20 °C. CO_2_ evolution was recorded with an infrared gas analyzer (URAS-2, Fa. Mannesmann, Düsseldorf, Germany). and calculated as mg CO_2_·h^−1^·kg.

The ethylene release was determined using the method as described by Vishnu Prasanna et al. [22]. Individual fruits of six replicates were enclosed separately in 4 L-tank glass jars for 1.0 h at 20 °C. Then the gas samples were drawn and C_2_H_4_ was measured in a Hewlett Packard GC (model 5890 series) fitted with Porapak Q column and FID detectors, using N_2_ as carrier gas. The gas concentration was calculated using standard C_2_H_4_ calibration gases in a HP 3396 series II Integrator and expressed as μL·kg^−1^ FW·h^−1^.

### 2.3. Preparation of Juice

Apples were organized into three batches of 10 fruits. For each batch, fruits were mechanically cut and then fruit pieces were randomly selected according to a systematic procedure. In order to reduce the browning of NFC apple juice during pressing, apple pieces were soaked in 0.5 g·L^−1^ ascorbic acid (food-grade) solution for 10 min, and then squeezed by a screw juicer (DAEWOO DY−BM03, Seoul, South Korea) [23]. After the apple juice was passed through a filter cloth of 200 mesh (74 µm), it was evenly mixed and packed into 200 mL transparent plastic bottles. The turbidity and particle size of some samples were measured immediately after treatment, and the remaining samples were quickly frozen with liquid nitrogen and stored at −80 °C. Before other quality indices were measured, they were thawed to room temperature in a water bath.

### 2.4. Determination of Juice Yield, pH, Titratable Acidity (TA), Total Soluble Solid (TSS), and Total Sugar

The juice yield of cloudy juice is found by calculating the percentage of cloudy juice quality of the total raw material quality of apple juice [17]. Meanwhile the pH of cloudy juice was measured with a pH meter (PHS−3E, Leisi Inc., Shanghai, China); 10 mL of fruit juice was diluted to 250 mL, titrated with 0.1 mol·L^−1^ NaOH solution by automatic potentiometric titrator (version D) to the end pH of 8.1, and then three parallel experiments were conducted for each sample. The volume of NaOH standard solution consumed was documented, and the final result was calculated as malic acid. The content of TA was calculated as shown in Formula (1):

(1) $$TA\%=\frac{V\times C\times K}{W}\times 100$$

where *V* denotes the volume of sodium hydroxide solution consumed by titration, mL; *C* is the concentration of sodium hydroxide solution, 0.1 mol·L^−1^; *W* stands for the weight of fruit juice, g; and *K* is the malic acid conversion coefficient, 0.067 [15].

TSS (◦Brix) was measured using a hand-held Brix refractometer (PAL-1, Pocket Inc., Tokyo, Japan). The concentrations of sucrose, glucose, fructose and sorbitol were determined utilizing high performance liquid chromatography (HPLC) with refractive index detection (RID). The mobile phase was HPLC grade water with a flow rate of 0.5 mL·min^−1^, while the separation column was Bio-Rad Aminex HPX-87C chromatographic column (300 × 7.8 mm; 9 μm particle size; Hercules, CA, USA). The column and detector temperatures were 80 and 35 °C, respectively. The true reference standard (Sigma-Aldrich, Burlington, MA, USA) of different volumes of sugar dissolved in water was injected to create a 5-point calibration curve. Cloudy juice samples were centrifuged (M-24A centrifuge, 12,000× *g*, 5 min) and hydrophilic PVDF injection filter (diameter 33 mm, pore size 0.45 μm; Merck, Kenilworth, NJ, USA) filtered the supernatant and then injected 10 μL. The total sugar is the sum of the amount of sucrose, glucose, fructose and sorbitol [24]. All measurements were carried out in triplicate.

### 2.5. Determination of Turbidity

Turbidity of juice samples was determined using the method proposed by Bhat and Goh [25] with only slight modification, in which 10 mL fruit juice was centrifuged at 4200× *g* at 25 °C for 10 min. Subsequently, the supernatant was collected and measured at 660 nm using a spectrophotometer (Spectra MAX 190, Biotek, Winooski, VT, USA), using distilled water as a blank. Turbidity (%T) is calculated as Formula (2), where Abs denotes the absorbance of supernatant at 660 nm [26].

Turbidity (T) = 100 − 100 × 10^−Abs^ (2)

### 2.6. Particle Size Distribution (PSD) Analysis

The PSD was measured by BT-9300ST Mastersizer (Better Instrument Co. Ltd., Dandong, China). The range it can measure extends from 0.1 to 2600 μm particles. The volume-mean diameter D (4,3), area-mean diameter D (3,2) and median particle diameter (D50) of all samples were calculated utilizing software (Microtrac-Bluewave, Montgomeryville, PA, USA) provided by the device. It was first necessary to clean the system with deionized water, then put the sample into a small-volume dispersion unit device and uniformly disperse it with a stirrer [27]. The measurement commenced when the ambiguity of the sample reached about 5%. All measurements were done in triplicate.

### 2.7. Determination of Starch

The starch content was determined by the procedure proposed by Carrin et al. [28]. In brief, a solution (126.9 mg·L^−1^) containing the same volume of 0.1 mol·L^−1^ iodine and potassium iodide (50 g·L^−1^) was diluted with cold distilled water to obtain a cold iodine solution (around 2 °C). An aliquot of 5 mL sample was mixed with 2.5 mL cold iodine solution, and after 10 min at 25 °C, followed by reading the absorbance at 615 nm with a spectrophotometer (Spectra MAX 190, Biotek, USA). The results were substituted into the calibration curves comprising corn starch solutions with different concentrations for the purposes of calculation. All measurements were conducted in triplicate.

### 2.8. Determination of Pectin 

Alcohol insoluble substances in apple juice were extracted according to Fraeye et al.’s [29] method, in which 50 mL of apple juice were mixed with 320 mL of 95% ethanol, stirred with a beater 3 times, each time for 6 s, and then subjected to suction filter to obtain precipitate. The precipitate was mixed with 160 mL of 95% ethanol again and this operation was repeated. Finally, it was rinsed with acetone to obtain a white and uniform powder, which was dried in a 40 °C oven to achieve a consistent weight. Determination of pectin content was carried out using a microplate reader; 5 mL of concentrated sulfuric acid-sodium tetraborate solution (0.015 mol·L^−1^) was added to 10 mg of ethanol insoluble matter, and immediately put into an ice-water bath to avoid the temperature rising too fast. After mixing with a mixer, it was put into a 100 °C water bath and heated for 5 min, then put into an ice-water bath immediately after the reaction until it reached room temperature. Then it was necessary to add 0.1 mL m-phenyl-phenol solution, and read the absorbance at 520 nm after mixing. Measurements were undertaken in triplicate.

### 2.9. Determination of Protein

Protein content was measured with a Coomassie Protein Assay Kit [30] (Solarbio Biological Technology Company, Beijing, China). In brief, protein was diluted to 2 g·L^−1^, then 10 μL mixed with 200 μL Coomassie Brilliant Blue (G-250), and reacted at room temperature for 10 min. With bovine serum albumin serving as the standard, the protein concentration was determined at the absorbance of 595 nm (Spectra MAX 190, Biotek, USA).

### 2.10. Determination of Total Polyphenol Content

The total polyphenol content was found utilizing the method established by Chan et al. [31], in which 1 mL of different juice sample were added to 2.5 mL 0.2 mol·L^−1^ Folin-Ciocalteu reagent respectively, and reacted in the dark for 5 min. Then, 2 mL of 7.5% (*w*/*v*) sodium carbonate were added to the mixture, stirred for 15 s and allowed to react in the dark for 2 h. Subsequently, the total polyphenol content was determined from the absorbance measured at 760 nm (Spectra MAX 190, Biotek, USA) and expressed as the equivalent content of gallic acid.

### 2.11. Statistical Analysis

All the experiments were evaluated by a completely randomized block in triplicate. We used one-way ANOVA to analyze the experimental data by IBM SPSS Statistics 19.0 (IBM, Armonk, NY, USA) and Duncan’s New Multiple Range. The objective was to establish the statistical significance (*p* < 0.05) test of the difference between the average values. Drawings were generated with Origin 8.5 software (OriginLab, Northampton, MA, USA).

## 3. Results

### 3.1. Analysis of Respiration Rate and Ethylene Release of Apple Fruit

**Figure 2:**
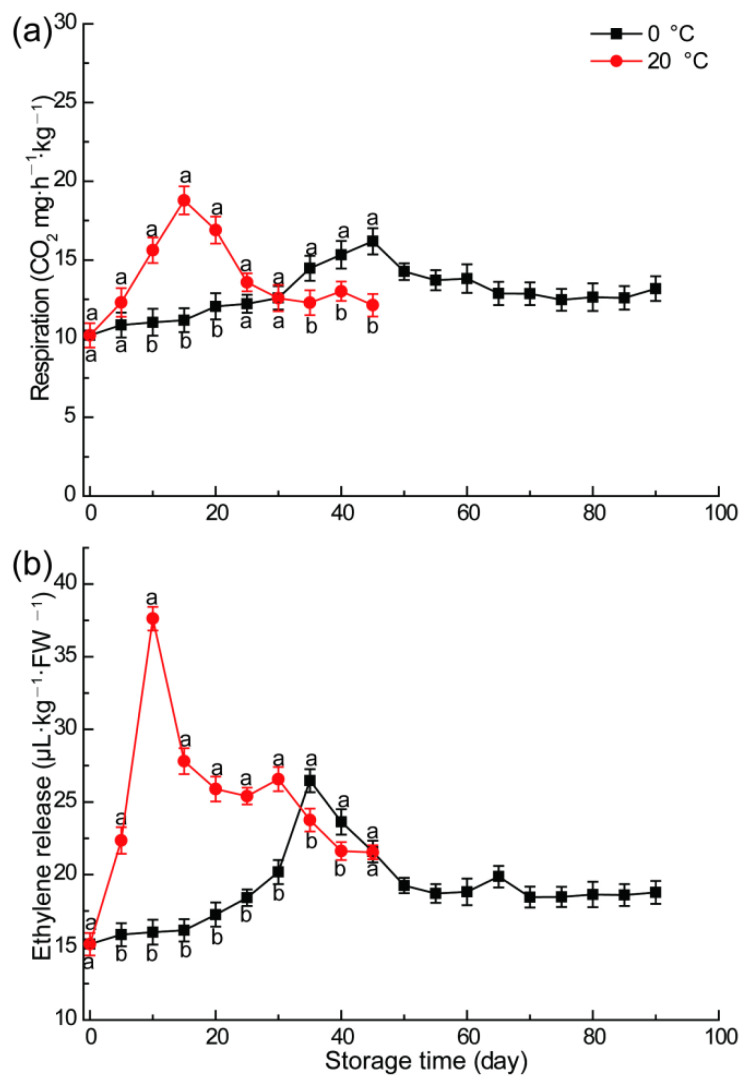
Respiration rates (**a**) and ethylene release (**b**) of apple fruit under different temperature and time. Vertical bars indicate ± standard errors of the mean values (*n* = 5). Different letters represent significant differences among different treatment for each sampling time at *p* < 0.05.

As a typical respiratory climacteric fruit, the respiratory rate of apple fruit significantly affects its physiological state [32]. As illustrated in Figure 2a, the respiratory rate of water-cored ‘Fuji’ apple increased rapidly after storage, reaching the peak of 18.78 CO_2_ mg·kg^−1^·h^−1^ after 15 days of storage, and then dropped speedily. Cold storage at 0 °C significantly reduced the respiration rate of fruits, and this gradually rose after storage. Potentially, the peak respiratory rate was delayed to 45 days after storage, delayed for 30 d, and the peak value was 16.18 CO_2_ mg·kg^−1^·h^−1^, which decreased by 13.8% compared with fruit storage at 20 °C. The results indicated that low temperature storage significantly delayed the respiration peak time of apple and reduced the peak value. This is basically consistent with the law of the respiratory rate of fresh-cut apples at different temperatures researched by Fagundes et al. [33]. It can be seen that low temperature can not only reduce the respiratory rate of fruit, but also the respiratory rate of fresh-cut slices. This is mainly because low temperature inactivate the enzymes related to physiological and energy metabolism such as respiration of apples, which significantly reduced the efficiency of respiration metabolism [34]. 

Compared with 0 °C cold storage, the ethylene release at 0 ◦C was significantly larger and increased faster (Figure 2b). It reached the first peak value of 37.62 μL·kg^−1^ FW·h^−1^ after 10 days and second peak value of 26.57 μL·kg^−1^ FW·h^−1^ after 30 days storage, respectively. Thereafter, with the fruit post-ripening and senescence, ethylene declined rapidly. Under the 0 °C cold storage scenario, the ethylene release increased slowly, and the ethylene release rate reached the first peak value of 26.46 μL·kg^−1^ FW·h^−1^ after 35 days and second peak value of 19.87 μL·kg^−1^ FW·h^−1^ after 65 days storage, respectively. Then it gradually diminished and maintained a low level. The peak of ethylene was delayed by 25 days, and the peak value decreased by 29.4% and 25.2%, which indicated that cold storage significantly reduced the peak value of ethylene and inhibited the production of ethylene. Because low temperatures can significantly reduce the release of ethylene, considering the ripening effect of ethylene [35], the post-ripening effect of apple fruit is greatly delayed, which also explained the reason why low temperatures delayed the peak of respiration. These results confirmed that storage temperature and time had very definite effects on respiration and ethylene physiological metabolism of apple fruit [34].

### 3.2. Analysis of Physical and Chemical Properties of NFC Apple Juice

Juice yield, pH, TA, TSS, total sugar and sugar/acid ratio are important physical and chemical indicators for quality control in NFC juice processing. As indicated in Table 1, the juice yields of the various storage temperatures and times varied. For fruit stored at 0 °C, the fruit stored for 45 days has the highest juice yield of 85.79%, whereas the fruit stored for 90 days had the lowest juice yield of 80.17%. For fruit stored at 20 °C, the fruit stored for 15 days had the highest juice yield of 84.78%, whereas the fruit stored for 90 days had the lowest juice yield of 78.23%, and the flesh of the water−core began to brown. This is basically consistent with the conclusion reached by Di Matteo et al. [36] in their analysis of lemon juice. Through our analysis, in the early stage of storage, the starch of fruit is gradually hydrolyzed into glucose, fructose and other monosaccharides with the post-ripening effect of apple [37], which made the juice yield of the fruit gradually increase, whereas the juice yield decreased in the late stage of storage due to the consumption of respiration and the increased water loss of the fruit due to aging [38].

Under the two storage conditions, the pH value of the cloudy juice squeezed from the fruit tended to increase (Table 1). The difference was that the pH of fruits stored at 0 °C was significantly lower than that of fruits stored at 20 °C (*p* < 0.05). This was positively correlated with the TA of NFC apple juice. When the storage period was extended, TA of apple juice revealed a downward trend, which may be due to the organic acids being converted into carbohydrates or oxidized during fruit post-ripening [39]. The end result was a decline in organic acids content.

TSS of fruit juice processed during fruit storage increased at first and then decreased (Table 1). During 20 days storage, the TSS increased in the first 15 days due to the hydrolysis of starch in fruit, while the starch was completely hydrolyzed in fruit after 15 days. It was noted that the consumption of substrate carbohydrates was dominated by respiration, so the TSS steadily decreased; 0 °C treatment significantly delayed post-ripening 30 days after respiration consumption. Consequently, a peak of TSS appeared at 45th day after the start of the experiment treatment [28].

Total sugar of the cloudy juice squeezed from the fruit revealed an decreasing trend and total sugar of fruits stored at 0 °C was slightly lower than that of fruits stored at 20 °C (Table 1). During storage, although some starch and organic acids were hydrolyzed and converted into sugar [37,39], this was not enough to counteract the consumption of respiration.

It has been reported that the sugar/acid ratio dictates the consumers’ preference for taste together with aromatic profile, and the balance between sweetness and sourness is very important in sensory experience [40]. Juice with high sugar/acid ratio (20 or higher) is usually favored by consumers, although some people prefer less sweet juice with a smaller sugar/acid ratio (<15) [41]. As demonstrated in Table 1, the sugar/acid ratio of juice was 20.58 at the beginning of the experiment, and increased steadily with the extension of storage time. By comparison, the juice from apples stored at 0 °C increased more slowly than that from apples stored at 20 °C. The results showed that the postharvest metabolic efficiency of organic acids in apple was higher than that of carbohydrates [39]. In the present study, the sugar-acid ratio of apple juice exceeded 20 from the beginning of storage. This maybe because our test materials-water-cored ‘Fuji’ apples came from Xinjiang with higher sugar and acid when harvested from trees [42].

### 3.3. Analysis of Turbidity of NFC Apple Juice

**Figure 3:**
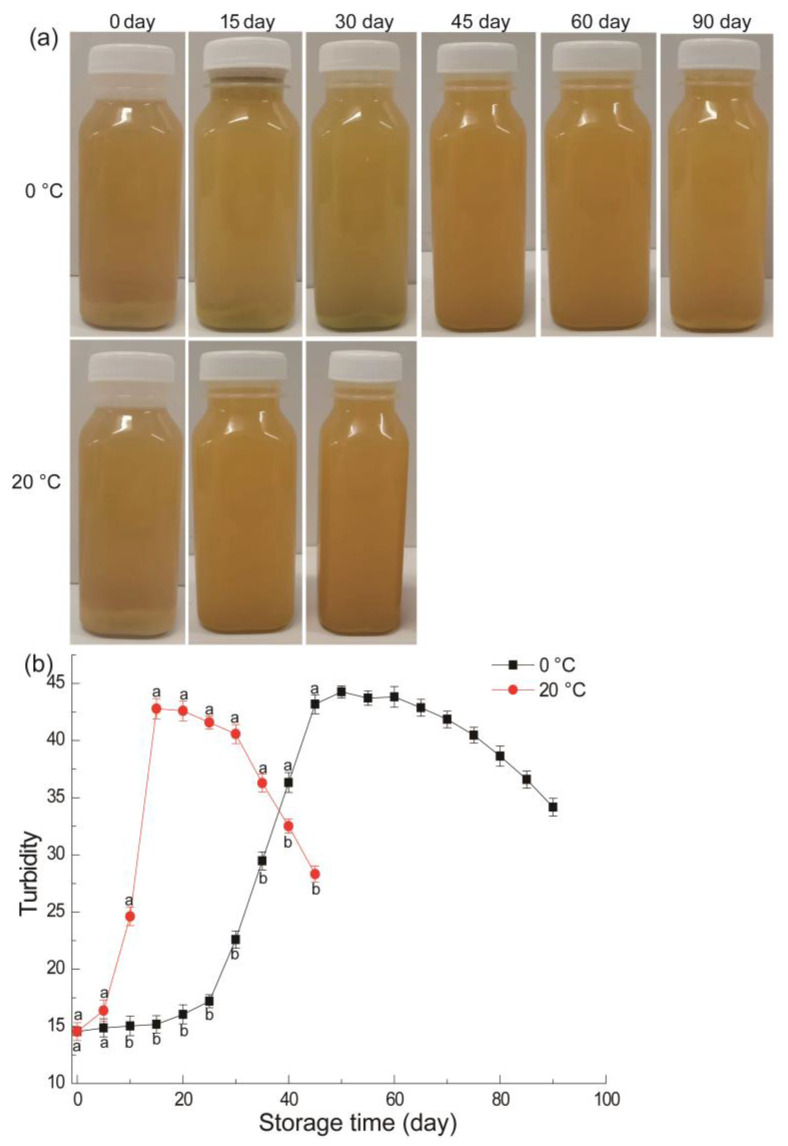
NFC apple juice come from fruit under different temperatures and times. Photograph (**a**), Turbidity (**b**). Vertical bars indicate ± standard errors of the mean values (*n* = 3). Different letters represent significant differences among different treatment for each sampling time at *p* < 0.05.

Turbidity directly reflects the stability of the juice system, and the larger its value, the more stable the juice system [43]. As demonstrated in Figure 3, the turbidity of apple juice varies significantly during different storage periods. For juice from fruit stored at 20 °C, fruits before storage showed the lowest turbidity (14.15) (Figure 3b) and the most sediment at the bottom of bottle (Figure 3a), whereas fruits stored for 15 days indicated the highest turbidity (45.22) and the least sediment at the bottom of bottle. After 30th day of storage, the turbidity of freshly squeezed juice dropped and the sediment rapidly increased at the bottom of the bottle. For juice from fruit stored at 0 °C, fruits stored for 45 days indicated the highest turbidity (46.88) and the least sediment at the bottom of bottle. The turbidity of freshly squeezed juice decreased and the sediment rapidly increased at the bottom of bottle during the last 30 days of storage.

As a typical climacteric fruit, the early storage period is the post-ripening stage in apple’s physiological process [44]. During this period, the residual starch in fruit is gradually hydrolyzed and converted into sugar, and the protopectin in the cell wall is gradually converted into water-soluble pectin [14]. With the peak of respiration, various invertases are constantly expressed and the protein content is constantly increasing [45], which is reflected in the fact that the turbidity of NFC juice is constantly increasing and the precipitation is constantly decreasing. In a study of Szczepańska et al. [46], turbidity of apple juice increased during 12 weeks refrigerated storage, which is basically consistent with the results of early storage in the present study. However, the present study has a longer storage time, so there is a significant difference in the late storage period. At the late stage of storage, pectin, protein and polyphenol in fruit decreased rapidly due to the ageing of the fruit [47], which resulted in the decrease of turbidity and the increasing precipitation in NFC juice.

### 3.4. Analysis of PSD of NFC Apple Juice

The pulp particles in cloudy apple juice are dispersed in the juice system in a suspended state. The suspension stability of cloudy apple juice was submitted to the Stokes sedimentation rate formula, and the particle size directly affects the sedimentation rate. The particle size of cloudy apple juice is proportional to the sedimentation rate, and the larger the particle size, the faster the sedimentation rate [1].

**Figure 4:**
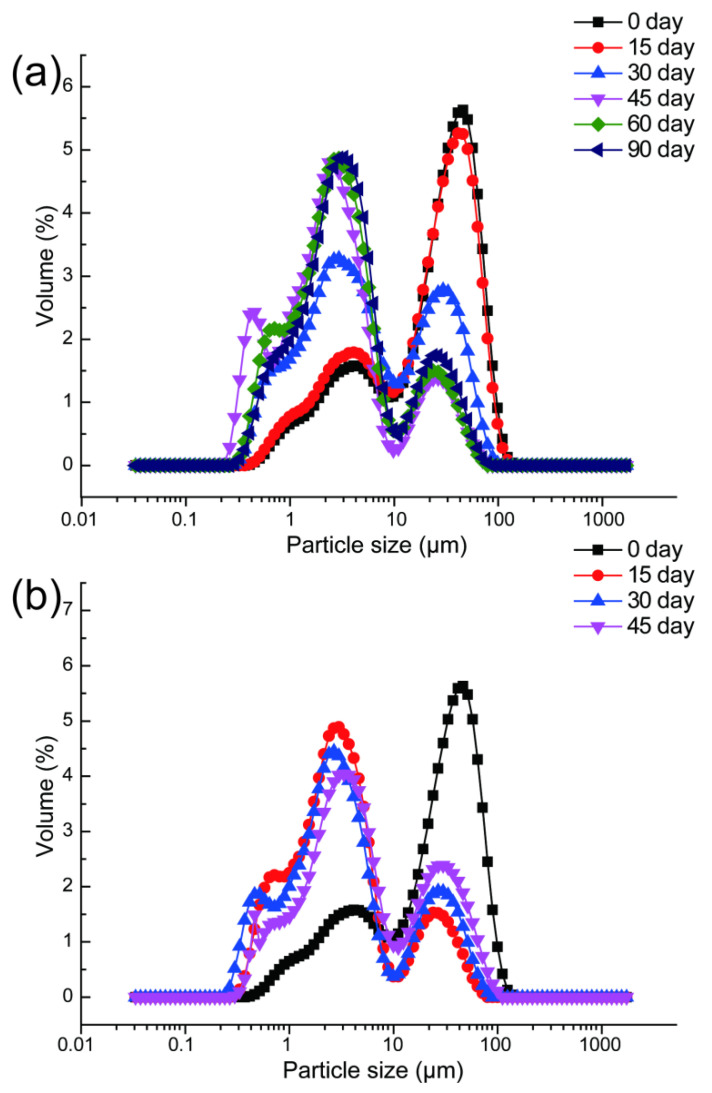
Particle size distribution (PSD) of NFC apple juice from fruit under different temperatures and times. 0 °C (**a**), 20 °C (**b**). (*n* = 3).

As indicated in Figure 4b, the change in the PSD of different NFC apple juices with post-ripeness and aging of fruit mainly presents a typical bimodal distribution under 20 °C storage. The larger particles were mainly distributed in 40~60 μm, whereas the smaller particles were mainly distributed in 2~3 μm in NFC apple juice. In early storage, the proportion of larger particles was higher than smaller ones in NFC apple juices. With the extension of storage period, the proportion of larger particles in NFC juice steadily declined, whereas the proportion of smaller particles increased significantly. After storage for 30 days, the proportion of smaller particles in juice was the largest, whereas that of larger particles was the smallest. However, when stored for 45 days, D50, D (3,2), D (4,3) of freshly squeezed juice were higher than that of 15, 30 days due to the ageing of fruits (Table 2). 

The PSD of freshly squeezed juices was similar to that of fresh juice treated at 0 °C and stored at 20 °C (Figure 4a). D50, D (3,2), and D (4,3) of freshly squeezed juices decreased continuously during the first storage period due to fruit post-ripening, and then increased after 60 days of storage due to aging of fruits (Table 2). The difference was that 0 °C cold treatment significantly delayed the whole process of fruit post-ripening and aging. This may be due to the continuous decomposition of the apple cell wall structure during post-ripening, which triggered the suspended particles in juice to become more dispersed and finer [48,49]. 

Previous studies have suggested that filtration [14], centrifugation [50], high pressure homogenization [9], high-intensity ultrasound [48], high pressure CO_2_ [51], and cold plasma [52] treatments have significant impact on the PSD of juice, and subsequently affect the turbidity and stability of juice. In the present study, analyzed from the perspective of raw materials, it was considered that the freshly squeezed juice from apples with peak respiration after harvest obtained the smallest D50, D (3,2) and D (4,3), and the best turbidity and stability [22].

### 3.5. Analysis of Sensitive Substances in NFC Apple Juice Precipitation

**Figure 5:**
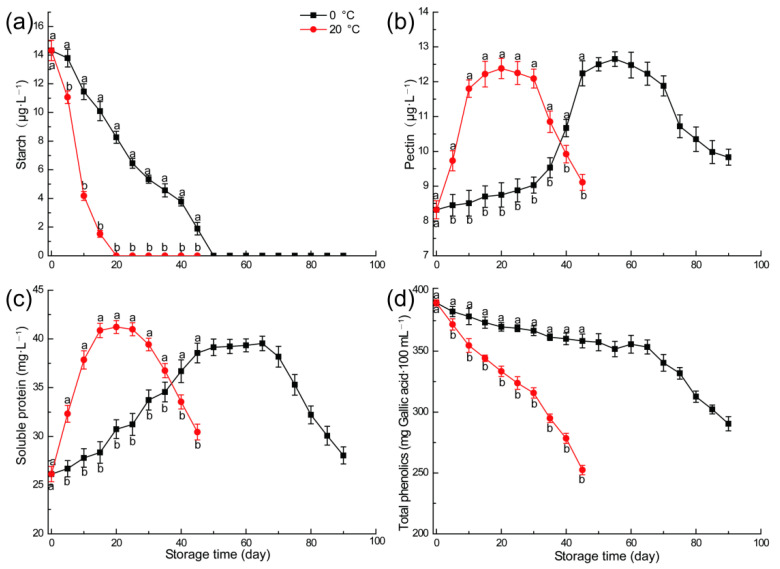
The concentration of starch (**a**), pectin (**b**), soluble protein (**c**), and total phenolics (**d**) in NFC apple juice from fruit under different temperatures and times. Vertical bars indicate ± standard errors of the mean values (*n* = 3). Different letters represent significant differences among different treatment for each sampling time at *p* < 0.05.

The main factors affecting the cloudy stability of apple juice are the interaction between molecules and aggregation of particles. The precipitated particles mainly contain polyphenols, protein and pectin [30], and insoluble starch is one of the main factors causing the precipitation of apple juice [28]. As depicted in Figure 5a, the starch in fruits was continuously hydrolyzed into fructose and glucose under the effect of post-ripening during storage. There were significant variations in starch content in apple juice from fruits stored at different temperatures and times. Therefore, in effect, the starch content decreased gradually during storage until it dropped to almost undetectable levels at the 20th day at 20 °C and 50th day at 0 °C, respectively. The starch in fruit was completely hydrolyzed into sugar nearby the peak of respiration, which was similar to the result of Vishnu Prasanna et al. [22]. These results were essentially consistent with the results of total sugar and sugar/acid ratio. In the early stage of storage, the total sugar and sugar/acid ratio decreased slowly before the peak of respiration, which was due to the hydrolysis of starch into sugar, thus offsetting part consumption of respiration. In the late stage of storage, starch has been completely hydrolyzed into sugar, and respiratory consumption dominated, so that the total sugar and sugar/acid ratio decreased rapidly [37].

Apple pectin plays an active role in maintaining the stability of cloudy juice, and can be wrapped outside the particles in the cloudy juice system, providing a protective coating for the interacting particles, thus improving the system’s stability [53]. As illustrated in Figure 5b, the apples just picked from trees contain large amounts of water-insoluble protopectin. Therefore, in early storage, with the extension of storage time, the apple gradually experiences more post-ripeness, and the original pectinase in the apple transforms the original pectin in the apple into water-soluble pectin, which helps to increase the stability of the juice system [54]. However, in the late storage period, pectin was degraded and consumed due to fruit ageing, though 0 °C cold treatment can delay and prolong the peak of pectin.

The polymerization of phenols and protein is considered to be an important factor causing the precipitation of apple juice [55]. The concentration of soluble protein in NFC apple juice originating from fruit under different temperatures and times is indicated in Figure 5c. The protein content of apple juice also increased at first, then decreased during the whole process, which was similar to the changes occurring in turbidity and pectin. This may be explained by the structure of apple pulp loosening with the increase in maturity, and the cell contents finding it easier to seep out under the mechanical force of juicing [56].

The total phenolics content of apple juices after picking revealed a downward trend during the whole storage process (Figure 5d). For fruits stored at 20 °C, the phenolics content fell significantly after 35 days of storage, while it decreased significantly after 70 days of storage due to ageing. This was similar to changes in the starch. The reduction in phenolic compounds leads to a diminishing of phenolic-protein polymer, which benefits the turbidity stability of apple juice [57].

## 4. Conclusions

Poor turbidity stability is an important factor restricting the progress of the NFC apple juice industry, and post-ripeness exerts a significant impact on turbidity stability. As a respiratory climacteric fruit, examples that have passed the peak of respiratory climacteric after being refrigerated and before ageing are more conducive to the turbidity stability of NFC juice. High proportion of small and medium-sized particles in juice are beneficial to helping retain stability. The influence of fruit storage temperatures and times on the turbidity stability of NFC apple cloudy juice is closely linked to the different composition and content of precipitation-sensitive substances (starch, total phenolics, soluble protein and pectin). When the starch and total phenolics exist in large amounts, the suspended system is unstable, whereas increasing pectin content can effectively improve the turbidity stability of juice. With continuous post-ripening, the insoluble starch in apples is gradually transformed into soluble monosaccharide, while the total phenolics content decreases and the pectin content increases. In short, appropriate pre-treatment helps to improve the turbidity and stability of apple NFC juice.

## Figures and Tables

**Table 1:** Juice yield, pH, titratable acids (TA), total soluble solids (TSS), and sugar/acid ratio (mean ± S.D.) for cloudy apple juice come from fruit storage under different temperature (0, 20 °C) and time (0, 9, 18, 30, 60 days).

Physiochemical Indexes	Storage Time/(day)	Storage Temperature/(°C)	
		0	20
Juice yield/(%)	0	80.76 ± 1.28 ^bc^	80.76 ± 1.28 ^bc^
	15	81.76 ± 1.61 ^b^	84.78 ± 1.58 ^a^
	30	82.86 ± 1.85 ^b^	82.39 ± 1.63 ^a^
	45	85.79 ± 1.32 ^a^	78.23 ± 1.68 ^c^
	60	84.56 ± 1.45 ^a^	-
	90	80.17 ± 1.56 ^a^	-
pH	0	3.91 ± 0.02 ^b^	3.91 ± 0.02 ^b^
	15	3.93 ± 0.03 ^b^	4.05 ± 0.02 ^a^
	30	3.93 ± 0.02 ^b^	4.08 ± 0.01 ^a^
	45	4.03 ± 0.02 ^a^	4.21 ± 0.03 ^a^
	60	4.05 ± 0.02 ^a^	-
	90	4.13 ± 0.03 ^a^	-
TA/(g malic acid·L^−1^)	0	0.57 ± 0.07 ^a^	0.57 ± 0.07 ^a^
	15	0.54 ± 0.06 ^a^	0.45 ± 0.04 ^b^
	30	0.51 ± 0.06 ^a^	0.44 ± 0.06 ^b^
	45	0.49 ± 0.08 ^a^	0.36 ± 0.06 ^c^
	60	0.44 ± 0.05 ^b^	-
	90	0.39 ± 0.04 ^c^	-
TSS/(°Brix)	0	13.63 ± 0.33 ^ab^	13.63 ± 0.33 ^ab^
	15	14.12 ± 0.24 ^a^	14.41 ± 0.29 ^a^
	30	14.12 ± 0.25 ^a^	13.82 ± 0.25 ^ab^
	45	14.54 ± 0.26 ^a^	11.11 ± 0.27 ^c^
	60	13.47 ± 0.23 ^ab^	-
	90	12.28 ± 0.31 ^b^	-
Total sugar/(g·L^−1^)	0	117.29 ± 0.67 ^a^	117.29 ± 0.67 ^a^
	15	116.23 ± 0.79 ^a^	115.23 ± 0.58 ^b^
	30	115.43 ± 0.81 ^b^	113.38 ± 0.78 ^bc^
	45	115.07 ± 0.87 ^b^	110.37 ± 0.91 ^c^
	60	114.96 ± 0.63 ^b^	-
	90	112.42 ± 0.78^c^	-
Sugar/Acid ratio	0	20.58 ± 0.96 ^d^	20.58 ± 0.96 ^d^
	15	21.52 ± 1.31 ^cd^	25.61 ± 1.45 ^b^
	30	22.63 ± 1.35 ^c^	25.77 ± 1.30 ^b^
	45	23.46 ± 0.75 ^c^	30.66 ± 1.52 ^a^
	60	26.15 ± 1.74 ^b^	-
	90	28.83 ± 1.95 ^ab^	-

Note: For values in a block, different lowercase alphabet letters indicate significant differences (*p* < 0.05) between means (*n* = 3).

**Table 2:** D (4,3), D (3,2), and D50 of cloudy apple juice (mean ± S.D.) from fruit storage under different temperatures (0, 20 °C) and times (0, 9, 18, 30, 60 days).

Stroage Temperature/(°C)	Particle Size/(μm)	Storage Time/(d)					
		0	15	30	45	60	90
0	D (4,3)	33.93 ± 0.13 ^a^	30.85 ± 0.15 ^b^	14.03 ± 0.22 ^c^	6.31 ± 0.01 ^g^	6.90 ± 0.08 ^f^	8.13 ± 0.09 ^e^
20	D (4,3)	33.93 ± 0.13 ^a^	6.94 ± 0.12 ^f^	8.89 ±0.17 ^e^	13.28 ± 0.28 ^d^	-	-
0	D (3,2)	6.13 ± 0.05 ^a^	5.34 ± 0.05 ^b^	2.13 ± 0.06 ^d^	1.15 ± 0.01 ^g^	1.56 ± 0.02 ^f^	1.82 ± 0.02 ^e^
20	D (3,2)	6.13 ± 0.05 ^a^	1.54 ± 0.01 ^f^	1.46 ±0.02 ^f^	2.20 ± 0.02 ^c^	-	-
0	D50	29.61 ± 0.15 ^a^	25.94 ± 0.16 ^b^	4.44 ± 0.12 ^c^	2.12 ± 0.03 ^g^	2.57 ± 0.05 ^f^	2.97 ± 0.04 ^e^
20	D50	29.61 ± 0.15 ^a^	2.52 ± 0.03 ^f^	2.54 ±0.03 ^f^	3.95 ± 0.06 ^d^	-	-

Note: For values in a block, different lowercase alphabet letters indicate significant differences (*p* < 0.05) between means (*n* = 3).

## Data Availability

The data that support the findings of this study are available from the corresponding author upon reasonable request.
